# Ecological genomics: steps towards unraveling the genetic basis of inducible defenses in *Daphnia*

**DOI:** 10.1186/1741-7007-8-51

**Published:** 2010-05-07

**Authors:** Ralph Tollrian, Florian Leese

**Affiliations:** 1 Department of Animal Ecology, Evolution and Biodiversity, Ruhr University Bochum, Universitaetsstrasse 150, D-44801 Bochum, Germany

## Abstract

Little is known about the genetic mechanisms underlying inducible defenses. Recently, the genome of *Daphnia pulex*, a model organism for defense studies, has been sequenced. Building on the genome information, recent preliminary studies in *BMC Developmental Biology *and *BMC Molecular Biology *have assessed gene response profiles in *Daphnia *under predation pressure. We review the significance of the findings and highlight future research perspectives.

See research articles http://www.biomedcentral.com/1471-2164/10/527, http://www.biomedcentral.com/1471-2105/6/45, http://www.biomedcentral.com/1471-213X/10/45

## Evolution of inducible defenses

Predation is a major selective force structuring biological communities and causing the evolution of defenses in many prey organisms. While permanent defenses evolve under constant predation pressure, inducible defenses are adaptations to heterogeneity in predation risk [1] and likely evolved under divergent selection regimes. Costs for the production or maintenance of defenses are saved during times when these defenses are not required. A fascinating aspect of the study of inducible defenses is that organisms with the same genotype can display dramatically different phenotypes in response to particular environmental factors that are required for activating genes that control the formation of these defenses. The interplay of genes and environment is far from being fully understood, above all since the genes and regulatory pathways have not been identified in most systems. The question whether 'plasticity genes' or interactions between multiple loci regulate plasticity, is an unresolved question (for example, [2]). Inducible defenses evolved in a range of evolutionarily diverse organisms from bacteria to vertebrates [1], but waterfleas (*Daphnia*), small planktonic crustaceans, are particularly famous for their large variety of spectacular inducible defenses. These inducible defenses include prominent morphological changes preventing capture [3,4], adaptive shifts in body size contrasting predator's prey-size selectivity [5], depth-selection behavior [6] to spectacular phenomena such as vertical migration where tons of zooplankton avoid predator encounters by changing their position in a diurnal rhythm.

## Model system *Daphnia*

**Figure 1:**
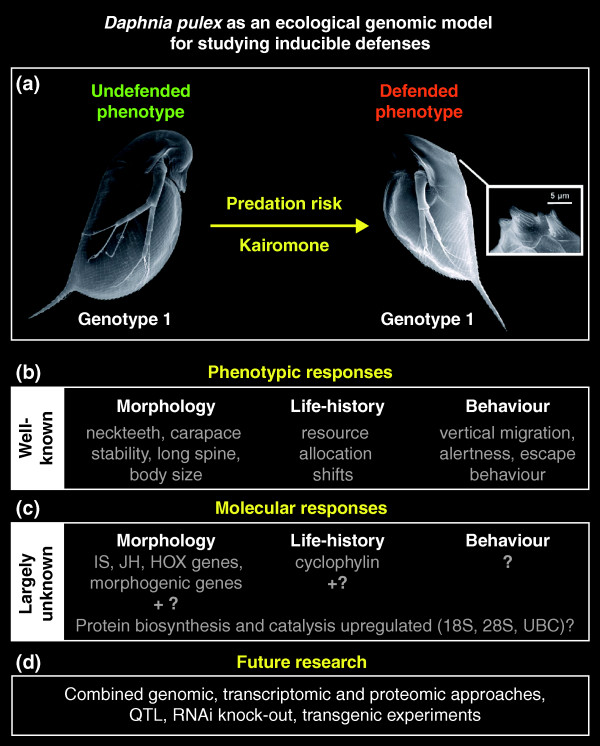
**Schematical representation of the current knowledge on inducible defenses in *Daphnia pulex***. **(a) ***Daphnia pulex *forms defenses in response to chemical cues (kairomones) released by predators. Even tiny 'neckteeth', morphological changes, are protective. **(b) **Whereas different phenotypic defenses (adaptive changes in morphology, life-history and/or behaviour) in response to predation pressure are reported from ecological studies, little is known about the genetic mechanisms underlying these phenotypic changes. **(c) **Preliminary evidence for an involvement of several genes in inducible defenses exists (IS, insulin signaling; JH, juvenile hormone; UBC, ubiquitin-conjugating enzyme). **(d) **Concerted multidisciplinary efforts are required in the future to address and answer the question about the genetic basis of inducible defenses. QTL, quantitative trait loci; RNAi, RNA interference.

Recently, the genome of *Daphnia pulex *has been sequenced and *Daphnia *is on the verge of becoming one of the first ecological genomic model organisms. Its ecology has been extensively studied over decades and this knowledge provides an advantageous contrast to most other model species. Furthermore, its cyclically parthenogenetic mode of reproduction allows studying genetically identical clonal lineages. *Daphnia pulex *show a number of defense strategies in response to chemical cues of predators, so called kairomones, chemical signals released by one organism and sensed by another to the disadvantage of the producer in this interspecific context (Figure 1).

## Finding relevant genes - candidate gene approach

An early pioneering study on a *Daphnia magna *lake clone has demonstrated drastic down-regulation of two proteins involved in basic cytoskeleton reorganization processes, alpha tubulin and actin [7]. With the availability of the v1.1 draft genome sequence of *Daphnia pulex *and numerous genomic tools, including microsatellite, cDNA, EST and cosmid libraries [8], it is now possible to link systematically the ecological information compiled over decades with the genomic data available for *Daphnia*. Building on the results of Pijanowska and Kloc [7], Schwarzenberger *et al*. [9] employed an *a priori *candidate gene approach using the genomic data available for *Daphnia pulex *to test for differential expression of several candidate genes in *Daphnia magna *in response to predator kairomones produced by fish and phantom midges. Interestingly, they found no significant changes to the expression level of alpha tubulin or actin genes in the same clone as used by Pijanowska and Kloc [7], hinting that posttranslational processes are involved in the effective decrease of the proteins. This study [9] did, however, provide evidence for strong upregulation of other genes in response to predator fish kairomone exposure, namely 18S and 28S rRNA genes involved in protein biosynthesis (2.2- and 1.9-fold); ubiquitin-conjugating enzyme (UBC), involved in protein catabolism (2.1-fold); and cyclophylin, involved in protein folding (2.9-fold). In the presence of phantom midge larvae (*Chaoborus *sp.) kairomone, 18S, 28S and UBC were likewise slightly upregulated (1.8-, 1.2- and 1.7-fold, respectively); however, cyclophylin was drastically downregulated (0.4-fold). This finding could hint at an involvement of cyclophylin in life history shifts since the responses in *Daphnia *differ for the two predator systems studied: for *Daphnia *under fish-predation the risks get smaller, while for those under *Chaoborus *predation it gets larger.

The most recent study, published in *BMC Developmental Biology *[10], addressed the question of what the regulatory genetic mechanisms are that underlie the inducible defense response. Miyakawa *et al*. [10] used both an *a priori*, candidate gene approach as in [9] and an *a posteriori*, differential display approach that does not require prior information on the genes to search for. They studied the expression of 31 candidate genes in late embryonic and first juvenile instar daphnids exposed to kairomone or to control media. Candidate genes were mainly those involved in endocrine, morphogenic and neuronal regulation based on earlier work, in particular on insect species. Emphasis was put on the juvenile hormone (JH) and insulin signaling (IS) pathways since studies on other arthropods have revealed that both seem to mediate the expression of morphogenic factors in the development of other phenotypically variable body appendages, such as beetle horns [11,12]. Candidate gene regions were identified in the *Daphnia pulex *draft genome sequence by homology searches. Transcriptional profiling by real-time quantitative RT-PCR (qRT-PCR) with newly synthesized primers worked for 29 of the 31 candidate genes. All 29 genes showed higher relative expression values (1.2- to 3.9-fold) in the kairomone-exposed first instar daphnids compared to the control animals when normalized to the traditional housekeeping gene *GAPDH *as a single internal reference gene. This up-regulation suggests that after kairomone reception, physiological changes through endocrine mechanisms, including the JH and IS pathways, are activated. This may lead to an upregulation of pattern formation and morphogenic genes.

## Finding relevant genes - differential display

In a second approach, Miyakawa *et al*. [10] used a whole genome scan technique, differential display (DD), to search for transcriptional differences among induced and non-induced daphnids also in the fourth embryonic and the first juvenile stages. The advantage of such genomic techniques is that no regions of interest have to be defined *a priori*, as compared to the candidate gene approach. DD yielded 22 differentially expressed candidate genes, for which three could subsequently be verified by real-time qRT-PCR as showing a moderate up-regulation in both embryos (1.3- to 1.4-fold) and juveniles (1.3- to 1.9-fold). Although no verified annotation of the three novel genes (*DD1*, *DD2 *and *DD3*) exists to date, homology searches performed by the authors predict functional roles for all three. *DD1 *was found to be the only gene with consistently higher expression in embryos as compared to juveniles. Database searches revealed the presence of a typical signal peptide and a dopamine beta-monooxygenase N-terminus domain (DOMON), typically involved in extracellular adhesion of receptors of yet unknown type. This finding leads to the speculation that this gene product may be involved in signal perception and/or transduction. The other two genes identified by differential display, *DD2 *and *DD3*, also lack strong homologies to eukaryotic genes and their functions remain the subject of speculation.

## Future perspectives

The studies by Schwarzenberger *et al*. [9] and Miyakawa *et al*. [10] present interesting first insights into the molecular genetic pathways primarily involved in morphological defense formation in *Daphnia pulex *and *Daphnia magna *under predation risk by *Chaoborus *larvae. A strength of the traditional deductive candidate approach employed in both studies is that it is based on a hypothesis about possible gene regulatory networks (for example, [11,12]). The finding of moderately up-regulated genes supports the hypothesis of the involvement of IS and JH and the expression of body pattern and morphogenic genes in the development of inducible defense structures. It is interesting to note, however, that although there is strong support for an involvement of JH, no JH receptor has been identified in the *Daphnia pulex *draft genome, despite considerable efforts.

The clear drawback of the candidate gene approach is its limitation to pathways and genes that are known. Novel pathways or genes relevant in this context may be missed completely. Hence, it is important also to scan for novel differentially expressed genes, as has been done by Miyakawa *et al*. [10]. Despite a large number of false positives or genes that did not amplify (19 of 22; H Miyakawa, personal communication) and despite the lack of clear homology for the remaining three genes (*DD1*, *DD2*, *DD3*), the detection of three intriguing novel genes highlights the potential of such genomic approaches for the discovery of novel transcripts that play an important role in induced defense formation.

One issue that will be important for future studies to address is how best to quantify changes in gene expression. The sensitivity of quantification in expression studies to different biases underlines the importance of validating expression levels of candidate genes using a great number of biological and also technical replicates. Since even classical housekeeping genes have been proven to be significantly differentially regulated in *Daphnia *[9,13], careful selection and evaluation of the internal reference genes for normalization of expression levels is important. If GAPDH is differentially regulated between kairomone-exposed *Daphnia *and the respective control, this may alter the interpretations of gene response profiles [13]. First results from tiling microarray data show that a large number of genes are potentially differentially regulated (JK Colbourne, personal communication). However, it is important to test with a greater number of biological and technical replicates to confirm up/down-regulation and, also for the microarray data, to assign levels of differential regulation, which can be considered as significant. In this context it should be kept in mind that as long as the kairomones have not been identified and a blend of different chemicals has to be used for induction, some genes might be activated as a side effect, that is, by compounds not related to the chemical alarm cues. Furthermore, predation is only one of several possible stressors to the organisms. Future comparative transcriptional profiling studies need to disentangle general stress responses from predation-specific responses.

With the advent of more sophisticated preparation techniques, tissue-specific expression profiles along a time course will greatly help in understanding fine-scale temporal and spatial gene expressions patterns. In this context, whole genome expression analysis by microarrays or RNA next-generation sequencing techniques will greatly facilitate studying these processes on a fine scale. Such studies should provide detailed insights into regulatory gene networks. Then, large-scale quantitative trait loci mapping, functional validation (at the protein level; Figure 1) and the subsequent extension to different *Daphnia pulex *clones and different *Daphnia *species should be performed to understand how predation signals are perceived, transmitted and refined into specific gene/protein regulatory pathways and subsequently into form and function. The fascinating array of inducible defenses manifested by *Daphnia *provides an interesting model for studying the interaction of genes with the environment. The application of new techniques in conjunction with genome sequence data is likely to yield significant new insights into the genetic basis of inducible defenses and phenotypic plasticty in general.
